# A Case of Filariasis: Removal of Lymphatic Varix by Operation

**Published:** 1907-09

**Authors:** J. Paul Bush, J. A. Nixon

**Affiliations:** Senior Surgeon to the Bristol Royal Infirmary; Assistant Physician to the Bristol Royal Infirmary.


### A CASE OF FILARIASIS: REMOVAL OF LYMPHATIC VARIX BY OPERATION.

BY

J. PAUL BUSH, C.M.G.,

*Senior Surgeon to the Bristol Royal Infirmary,*

AND

J. A. NIXON, M.B., M.R.C.P.,

*Assistant Physician to the Bristol Royal Infirmary.*

---

In October, 1906, a negro, aged 19, was admitted to the Royal Infirmary under Dr. Nixon on account of elephantiasis. His home had been in Demerara, where his parents, two brothers and four sisters are still living and healthy; one brother has died from "diarrhœa." Previous to leaving Demerara he had suffered from occasional attacks of fever, but during the last five years these had been absent. He came to England two-and-a-half years ago, and has followed many occupations—ship's steward, professional boxer, and motor-car cleaner. About six months after his arrival two abscesses appeared on his right leg; they burst and healed spontaneously without further trouble.

About seven months before he came under our observation, a swelling in the left groin began to trouble him—not by pain, merely by the discomfort of its presence; its growth was slow but steady. A month after the swelling appeared in the groin he noticed that the left leg as a whole was increasing in size quite painlessly; he was also struck by the fact that both the general fulness of the leg and the localised tumour in the groin increased on standing and decreased after resting. On one occasion he intentionally pricked the leg with a needle, and a milky fluid escaped, but the puncture healed in a few days.

*On admission* the patient presented all the appearances of vigorous health and considerable physical development. The heart and lungs showed nothing abnormal. The pulse rate was 88, respirations 24, and the temperature was 97° F. His abdomen was soft, flaccid, and not distended; the liver and spleen were not enlarged. In the left groin, filling Scarpa's triangle and apparently outlined by it, was an elastic, irregularly-lobulated swelling neither painful nor tender; above it was very definitely limited by Poupart's ligament. At first sight, and to the touch, it represented a soft lipoma, but there was a marked impulse on coughing. It had an ill-defined feeling of fluctuation, and by pressure on one half could be almost completely made to disappear in the part under compression, with a corresponding increase of fulness in the other part; it felt, in fact, like a wet sponge in an india-rubber bag. When the patient lay down for a short while the tumour became obviously smaller, and then irregular knotted cords could be felt traversing its substance; firm pressure on the abdomen just above Poupart's ligament conveyed the impression of a similar fulness in the pelvis on that side, which was absent on the right; no enlarged glands could be made out. The thigh was not much increased in size, but below the knee there was considerable œdema, with the characteristic hardness and ruggedness of the skin which is associated with elephantiasis. At the middle of the calf the girth of the left leg was four inches greater than the right; the skin was not broken or inflamed. There was no sign of lymph scrotum, or of any varicosity of lymphatics in other regions.

On the night subsequent to that of his admission the patient had a rigor, and his temperature rose to 102°; the attack was by no means a complete or definite "ague-fit," and never recurred at any time. The temperature remained constantly normal afterwards.

Mr. Rudge, our house physician, examined the blood, and found the filaria sanguinis hominis present at nights; the results of his investigations are appended to this report in their entirety. The swelling in the groin was explored shortly after admission with a hypodermic syringe; no fluid entered the syringe, but on withdrawing the needle, a drop of milky fluid appeared at the seat of puncture; by pressure a considerable amount of chylous fluid was procured, but on examination no filariæ were found (the time of puncturing was 3 p.m.). Rest in bed and the application of pressure made no material difference to the condition of the leg.

It was then represented to the patient that an operation for the removal of the tumour might be successfully undertaken, but that the cure of his elephantiasis or the discovery of the parent worm or worms (in whose life history he took a keen interest) were improbable, the chylous nature of the fluid making it unlikely that the actual point of obstruction was low enough down to be accessible. Understanding the circumstances, he decided that he would have the operation performed after Christmas, and returned home for a month.

On January 16th, 1907, Mr. Bush operated for the removal of the lymphatic varix. Turning up a large triangular flap of skin in the groin, he exposed the mass, ligatured the larger lymphatics above and below as they entered the varicose plexus, divided the internal saphena vein which was buried in the mass, and then cut through its remaining attachments, excepting an extension of the plexus which was found to pass through the saphenous opening, communicating probably with similarly dilated lymphatics more deeply situated. This was ligatured and divided, the varix when removed being about large enough to fill a soup plate (the lymph contained in its spaces having for the most part escaped) ; no trace of the parent worm was found in it.

So far as was possible all oozing points in the area laid open were ligatured before closing the wound, and a drainage tube was inserted. No attempt was made to follow the lymphatics into the pelvis and remove them from that situation, into which they obviously extended.

The patient made an uneventful recovery, and left the Infirmary a month after the operation ; the wound was not completely healed, and there was still a slight leaking of lymph. The general swelling of the leg was somewhat less, and the patient expressed himself as much more comfortable since the lump in his groin had been removed. As he no longer resides in Bristol, we are not aware whether the discharge of lymph has stopped and the wound healed.

The appearance of the filaria in the blood was in no way affected by the operation ; the embryos were found at the usual time during the night in undiminished numbers from the date of operation to the time of discharge.

Since the publication of Cunningham's monograph (with its extensive bibliography)[1] it seems of great importance that cases of filariasis which are submitted to surgical treatment should be reported in full, so that evidence may be amassed upon which sound conclusions may ultimately be based. For at present, if surgery fails to relieve the subjects of filariasis, they lie indeed under no obligation to "internal medicine."

[1] *Ann. Surg.*, 1906, xliv. 481.

BLOOD REPORT BY F. H. RUDGE, M.R.C.S., L.R.C.P.

The examination of the blood of this patient has not demonstrated any new facts as to the characteristics or life-history of the filaria nocturna embryo. The presence or absence of the embryo in the peripheral blood taken at various times during the day and night is indicated in the appended table :

| | | | |
|---|---|---|---|
| 12 midnight | numerous. | 12 noon .. | none. |
| 2 a.m. .. | present. | 4 p.m. .. | none. |
| 4 a.m. .. | present. | 6 p.m. .. | none. |
| 5 a.m. .. | present. | 7 p.m. .. | none. |
| 6 a.m. .. | few. | 8 p.m. .. | present. |
| 7 a.m. .. | none. | 9 p.m. .. | present. |
| 9 a.m. .. | none. | 10 p.m. .. | numerous. |
| 11 a.m. .. | none. | 11 p.m. .. | numerous. |

The advent of the embryos appears to be very rapid, as indicated by the fact that while at 7.30 p.m. none could be found, at 8 p.m. they were fairly numerous. Their disappearance was equally abrupt. The number, which average about twelve in a drop of blood equal to two minims, was greatest between the hours of 10 p.m. and 2 a.m. On one occasion a solitary embryo was found at 10.30 a.m. It was in every respect similar to those found during the night, except that it was not so active. This was after the lymphatic mass in the groin had been removed by operation. This was the only occasion when the worm was found during the day, and may be regarded as purely accidental.

It is unusual to find two or more in close proximity ; in fact, they appear to be particularly prone to remain aloof from each other. Cold soon kills them, and their habit of leaving their sheaths under its influence was frequently demonstrated. If kept at body temperature, they would remain alive even under a cover-slip for forty-eight hours. An attempt to stain them *in situ* was made by giving the patient methylene blue pills, but although this was continued for a week, it was of no avail. The nuclei of the leucocytes were stained lightly. Empty sheaths were frequently found, and were noticed to be more numerous when the live embryo was not present.

The result of the blood-counts was as follows :—

| | 12 *noon.* | 12 *midnight.* |
|---|---|---|
| Red .. .. | 8,552,000 | 6,720,000 |
| White .. | 10,600 | 12,200 |
| | PER CENT. | PER CENT. |
| Polymorphonuclear .. | 59.8 | 53.24 |
| Large mononuclear .. | 14.6 | 12.23 |
| Small mononuclear .. | 9.4 | 17.02 |
| Eosinophiles .. .. | 12.8 | 13.29 |
| Basophiles .. .. .. | 2.5 | 4.35 |

This result only bears out what has already been described, namely the presence of eosinophilia. It seems questionable whether the increase in the eosinophilia when the worm was present, though constant, is a point from which one can draw any inference. If it were more marked, one might reasonably assume that the eosinophiles had a phagocytic action on the embryos. The counts were made frequently, but the above results are representative.

A microscopical examination of the chylous fluid obtained by aspiration of the mass in the groin demonstrated that the filaria was absent.

